# Supplementary material for: Barriers and facilitators to vaccination uptake against COVID-19, influenza, and pneumococcal pneumonia in immunosuppressed adults with immune-mediated inflammatory diseases: A qualitative interview study during the COVID-19 pandemic
Source: PLoS One. 2022 Sep 9;17(9):e0267769. doi: 10.1371/journal.pone.0267769 (PMC9462800; doi:10.1371/journal.pone.0267769)
Supplement: S1 Table — (DOCX) [file pone.0267769.s001.docx]

**Table S1. Participant characteristics.**

| Female gender, n (%) | 15 (75) |
| --- | --- |
| Non-white ethnicity, n (%) | 4 (20) |
| Age in years, range | 27-72 |
| Vaccine uptake, n (%)  Always  Sometimes  Often not | 6 (30)  7 (35)  7 (35) |
| Diagnosis, n (%)  Rheumatoid arthritis  Crohn’s disease or Ulcerative colitis  Vasculitis  Systemic Lupus Erythematous  Ankylosing Spondylitis/psoriatic arthritis | 5 (25)  4 (20)  4 (20)  3 (15)  4 (20) |
| Biologic medication, n (%) | 13 (65) |
| At-risk factors, n (%)  Aged >=65 years  Asthma  Diabetes  None | 3 (15)  6 (30)  2 (10)  9 (45) |
| Location, n (%)  Midlands  London and the South  Rest of the UK | 10 (50)  7 (35)  3 (15) |
