# Supplementary material for: Barriers and facilitators to vaccination uptake against COVID-19, influenza, and pneumococcal pneumonia in immunosuppressed adults with immune-mediated inflammatory diseases: A qualitative interview study during the COVID-19 pandemic
Source: PLoS One. 2022 Sep 9;17(9):e0267769. doi: 10.1371/journal.pone.0267769 (PMC9462800; doi:10.1371/journal.pone.0267769)
Supplement: S1 Methods — (DOCX) [file pone.0267769.s002.docx]

**S1 Supplementary methods. Participant eligibility criteria and recruitment.**

Eligible conditions: rheumatoid arthritis, inflammatory bowel disease, psoriasis +/- arthritis, ankylosing spondylitis, vasculitis (small vessel or large vessel), systemic lupus erythematosus, or reactive arthritis.

Eligible immune-suppressing medication: methotrexate, azathioprine, 5-mercaptopurine, sulfasalazine, mycophenolate mofetil, leflunomide, ciclosporin, tacrolimus, or sirolimus, biologic agents.

Recruitment details: Potentially eligible patients were sent study information by their usual care team (NUH) or via patient charity’s newsletters, website and social media accounts. National Rheumatoid Arthritis Society, National Ankylosing Spondylitis Society, Crohns and Colitis US, Vasculitis UK, Lupus UK and Psoriasis Association UK supported the recruitment. Those willing to participate were asked to complete a reply slip (see below) either online or in paper form, which asked them to self-report age, sex, ethnicity, physician-diagnosed inflammatory condition(s), comorbidities, and engagement with vaccination. Participants confirmed their immunosuppressing medication at the interview.

Reply slip questions:

| 1. I am interested in taking part in this study *(Yes / no)* 2. How old are you? (Years) 3. What is your gender? (Male / female / other (please specify)) 4. What is your ethnicity? (White / mixed/multiple ethnic groups / Asian or Asian British / Black/African/Caribbean/Black British / Other (please specify)) 5. Have you been diagnosed with any of the following? (Rheumatoid arthritis / psoriatic arthritis / systemic lupus erythematosus / ankylosing spondylitis / vasculitis / Crohn’s disease or ulcerative colitis / other (please specify)) 6. Have you been diagnosed with any of the following? (Diabetes / asthma / dementia / COPD, also called emphysema or chronic bronchitis / none of the above) 7. Have you had the pneumonia vaccine? (Yes / no) 8. Have you had a flu vaccine? (Yes / no) 9. If yes, how many times in the past three years have you had the flu vaccine? (None, it was more than three years ago / once / twice / three times or more) 10. Have you had the COVID-19 vaccine? (Yes, both / Yes, one and I will have the second / Yes, one but I am not having the second / No, but I intend to / No and I do not intend to) |
| --- |
