# Supplementary material for: Barriers and facilitators to vaccination uptake against COVID-19, influenza, and pneumococcal pneumonia in immunosuppressed adults with immune-mediated inflammatory diseases: A qualitative interview study during the COVID-19 pandemic
Source: PLoS One. 2022 Sep 9;17(9):e0267769. doi: 10.1371/journal.pone.0267769 (PMC9462800; doi:10.1371/journal.pone.0267769)
Supplement: S2 Methods — (DOCX) [file pone.0267769.s003.docx]

# S2 Supplementary methods. Interview guide questions.

| **Risk perceptions of flu and pneumonia** | 1. What is your understanding of flu? What is your understanding about the risk it poses to your health as someone with an inflammatory condition?   Repeat for pneumonia. |
| --- | --- |
| **Vaccination knowledge and information – flu and pneumonia** | 1. What is your understanding of vaccinations and how they work?   If participant has not had the pneumonia vaccine:   1. Are you aware of needing the pneumonia vaccination? Have you ever been offered the pneumonia vaccination?   Prompt: conversations with any healthcare professional about the pneumonia vaccine, or received information about it.  If participant has had the pneumonia vaccine:   1. Tell me about your experience of being offered the pneumonia vaccination?   Prompt: conversations with any healthcare professional.   1. What information were you given about why you were offered this vaccination? 2. Have you ever seen or found information about pneumonia vaccination anywhere else?   Prompt: through social media, messaging services.  Repeat for seasonal flu. |
| **Vaccination attitudes and experiences – flu and pneumonia** | 1. Can you tell me about how you decided to have / to not have / to have some but not all of these vaccinations?   Prompt: concerns / benefits to having these vaccinations.  If not mentioned: having IMID or impact of vaccination on it / Being on immune-suppressing medication (or impact of vaccination on it / Understanding of need to be vaccinated / Social network / Confidence in the safety of the vaccine / Perceived risk of respiratory illness / Ability to access vaccination services and the suitability of them (cultural, disability, communication, time considerations).   1. Have you ever discussed being vaccinated for the flu or pneumonia with anyone else who is not a healthcare professional?   Prompt: role they had in deciding to have these vaccinations.   1. What was the most important factor in making your decision to have / not have some / all of the pneumonia and seasonal flu vaccinations? |
| **Vaccination knowledge, attitudes and experiences – COVID-19** | 1. What is your understanding of Covid-19? What is your understanding about the risk it poses to your health as someone with an inflammatory condition? 2. Has the Covid-19 pandemic changed your views on vaccinations in any way?   Prompt: whether you will accept seasonal flu/pneumonia vaccinations in future, the importance of being vaccinated. What prompted this change?  Then repeat questions 4-9 for COVID-19.  **We have come to the end of the interview, do you have any further comments you would like to make about what we have discussed today? Thank you for your time.** |
