# Supplementary material for: Barriers and facilitators to vaccination uptake against COVID-19, influenza, and pneumococcal pneumonia in immunosuppressed adults with immune-mediated inflammatory diseases: A qualitative interview study during the COVID-19 pandemic
Source: PLoS One. 2022 Sep 9;17(9):e0267769. doi: 10.1371/journal.pone.0267769 (PMC9462800; doi:10.1371/journal.pone.0267769)
Supplement: Attachment — Submitted filename: Plos.docx [file pone.0267769.s004.docx]

The manuscript is well written and well timed as even in the midst of pandemic vaccine refusals have been rampant and unfortunately so even in the immunocompromised group.

Multiple quantitative studies have been published on the topic of vaccine hesitancy but qualitative studies are rarer especially in the patient population of inflammatory immune mediated diseases. Gives more insight in the thought process of the actual patients.

It is interesting that patients actually prefer using patient resource centers and dedicated websites for vaccine information over social media websites. At the same time advertisements at GP offices actually encouraged vaccination uptake. So it’s the different type of media that is viewed differently by patients when it comes to making informed health decisions.

This study brings out a fact that patients with chronic disease tend to rely on their specialists even for basic primary care like vaccination and there appears to be an underlying bias that specialist’s opinion about vaccination is more trustworthy. While in clinical practice primary care physician can play an important role in increasing vaccine awareness and receptiveness. Yearly checklists of things to be done for health maintenance is one of the major work arenas of primary care.

It is interesting to note that there is a difference from the Canadian study where there was mistrust about pharmaceutical companies which was not seen in this UK based study. It opens an arena about are there differences in vaccine uptake and vaccine hesitancy.

Discussing the above points in a little more detail might improve the manuscript even more. Overall, a well written and thought about paper
