# Supplementary material for: Barriers and facilitators to vaccination uptake against COVID-19, influenza, and pneumococcal pneumonia in immunosuppressed adults with immune-mediated inflammatory diseases: A qualitative interview study during the COVID-19 pandemic
Source: PLoS One. 2022 Sep 9;17(9):e0267769. doi: 10.1371/journal.pone.0267769 (PMC9462800; doi:10.1371/journal.pone.0267769)
Supplement: Attachment — Submitted filename: Approval of data availability statement.docx [file pone.0267769.s005.docx]

*27 July 2022*

*Academic Rheumatology,*

*Clinical Sciences Building*

*Nottingham City Hospital*

*Nottingham NG5 1PB*

*Email:* [*amy.fuller@nottingham.ac.uk*](mailto:amy.fuller@nottingham.ac.uk)

*Tel: 0115 823 31761*

Dear Freddie Domini,

PLOS ONE

Thank you for sending the drafted Data Availability statement below. I can confirm this information is accurate and approve this statement to be used in the paper.

*"Transcript excerpts relevant to the study are included within the paper, and the interview guide has been included as a Supporting Information file. Additional qualitative data and audio recordings are archived in the University of Nottingham servers using password protection, and are restricted due to privacy concerns. Data are available upon request from the Research Governance team (*[*sponsor@nottingham.ac.uk*](mailto:sponsor@nottingham.ac.uk)*) for researchers who meet the criteria for access to confidential data."*

Yours sincerely

Dr Amy Fuller,

On behalf of all authors
